# Trastuzumab holds potential to accelerate spontaneous sensory reinnervation after free flap breast reconstruction: a proof of concept

**DOI:** 10.1515/iss-2023-0070

**Published:** 2024-07-24

**Authors:** Jana Leskovar, Marko Petrovečki, Krešimir Bulić

**Affiliations:** University of Zagreb School of Medicine, Zagreb, Croatia; Department of Radiology, University Hospital Centre Zagreb, Zagreb, Croatia; Department of Surgery, University Hospital Centre Zagreb, Zagreb, Croatia

**Keywords:** free tissue flaps, peripheral nerve injuries, sensation, trastuzumab

## Abstract

**Objectives:**

Breast sensation following autologous breast reconstruction impacts patients’ quality of life. Although spontaneous reinnervation in free flaps was documented by many authors, there are efforts to further improve restoration of breast sensation. Interestingly, animal studies indicated that trastuzumab has several beneficial effects on transected peripheral nerves. Our aim was to compare spontaneous sensory recovery after free TRAM flap breast reconstruction between patients who were and were not treated with trastuzumab.

**Methods:**

The study included 14 subjects who underwent tactile sensation examination in 5-year period after noninnervated free muscle-sparing TRAM flap breast reconstruction at the University Hospital Centre Zagreb, Croatia. Small and large flap skin islands and contralateral healthy breasts were tested with Semmes-Weinstein type monofilaments. Three sensory scores were created to more accurately compare breast sensation.

**Results:**

In subjects receiving trastuzumab, sensory recovery earlier extended to at least four of five large skin island regions and was always present in the central flap area in comparison with subjects who were not administered trastuzumab (p=0.0476). As indicated by total sensory scores, trastuzumab-treated subjects restored sensation better resembling healthy control breasts (54 vs. 39 % in large skin islands; 95 vs. 71 % in small skin islands).

**Conclusions:**

To the authors’ knowledge, the current study for the first time demonstrated trastuzumab’s potential to improve sensory outcomes in human. Our results support the strategy that accelerated nerve regeneration is a key to more successful reinnervation. HER2 and EGFR inhibitors emerge as new candidates for pharmacological interventions in peripheral nerve injury treatment.

## Introduction

Spontaneous sensory recovery following autologous breast reconstruction with noninnervated flaps was documented by many authors [1], [2], [3], [4], [5], [6], [7], [8], [9], [10]. Aspirations toward improved sensation had led to the idea of innervated breast reconstruction involving nerve repair that was first actualized by Slezak et al. in 1992 [11]. Since then, a number of authors have been applying different nerve coaptation techniques to various recipient and donor nerves aiming to achieve superior sensory restoration [4], [10], [11], [12], [13], [14], [15], [16], [17], [18]. The studies directly comparing innervated to noninnervated free TRAM (transverse rectus abdominis myocutaneous) or DIEP (deep inferior epigastric perforator) flaps have demonstrated significantly better recovery of multiple sensory modalities [14], tactile thresholds [12], 16], 17], and temperature discrimination [13] in innervated flaps. Although the sensation levels of healthy control breasts were not reached [14], increased objective tactile sensation accomplished in innervated free flaps was found to be associated with improved patient-reported quality of life [16], 18].

Roughly one in five women diagnosed with breast cancer has HER2-positive disease [19]. HER2 stands for the human epidermal growth factor receptor 2 [20], which aberrant expression is related to adverse prognosis in breast cancer [19]. Patients who meet HER2-positivity criteria are eligible for HER2-targeted therapy [21]. Trastuzumab (HERCEPTIN, Genentech, South San Francisco, CA) is a recombinant humanized monoclonal antibody directed at HER2 antigen [20]. Acting as a tumor-targeting monoclonal antibody, it is a form of passive anticancer immunotherapy [22]. Trastuzumab is approved as a component of the treatment regimens for HER2-positive metastatic and early breast cancer [20].

Animal studies on peripheral nerve regeneration described several novel actions of trastuzumab [23], 24] most evident being enhancement of axonal sprouting [23]. In particular, their results showed that trastuzumab was able to increase a number of regenerated myelinated axons [23], 24] as well as a number of motor and sensory neurons emitting their axons and to encourage Schwann cells and macrophages proliferation in distal nerve stumps [24].

Our cross-sectional study was originally conducted to evaluate spontaneous recovery of sensation in 5-year period after noninnervated free flap breast reconstruction. During the careful analysis of the results, an unexpectedly faster and more consistent recovery of tactile sensation was found in patients treated with trastuzumab. For the purpose of this article, the data were re-processed aiming to imply the probable positive effects of trastuzumab on peripheral nerve regeneration in human.

## Materials and methods

All patients who underwent noninnervated free microvascular muscle-sparing (MS) TRAM flap breast reconstruction between January 2014 and November 2018 at the University Hospital Centre Zagreb, Croatia were invited to enter the study. Examinations were done during a single visit to our Department of Plastic Surgery independently of the routine follow-up, which is usually completed 6 months post-reconstruction. All enrolled patients signed an informed consent. Ethical approvals were obtained by the Institutional Ethics Committees of the University Hospital Centre Zagreb and the University of Zagreb School of Medicine.

### Subjects’ classification and characteristics

Subjects were classified into two main experimental groups according to the flap skin area size: small skin island and large skin island group. Each of them was then subdivided into IMMUNO+ and IMMUNO− subgroups based upon a history of immunotherapy administration. The unoperated contralateral breasts were considered healthy controls. To make the reference breast as representative as possible, two control groups were formed for each of the main groups. To further characterize our subjects, data on entire breast cancer treatment, age, body mass index, and comorbidities were collected through the medical records.

### Sensory testing

**Figure 1:**
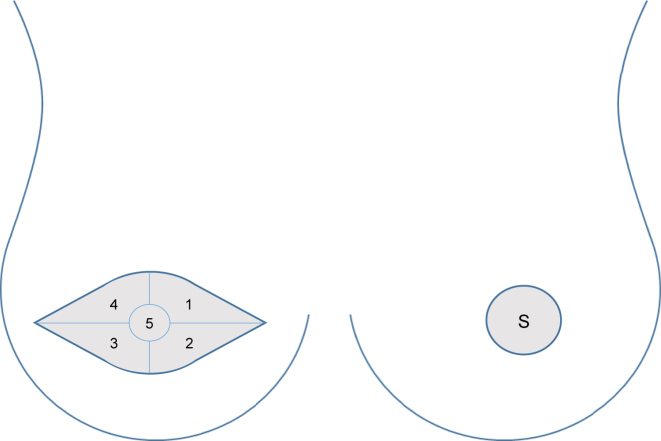
The appearance of large skin island on the left and small skin island on the right. Light gray areas depict exposed skin of the flaps. In large skin island, the following regions were tested: 1 – superior medial, 2 – inferior medial, 3 – inferior lateral, 4 – superior lateral, and 5 – central. Small skin island was tested as an unique area herein marked with an S.

To estimate and describe tactile function of the flap skin, Semmes-Weinstein monofilament hand-held test was employed [25]. Sensory testing was performed solely on exposed skin of the flaps. As already explained, flaps with small and large skin islands were discerned. Small skin island denotes the residual flap skin shaped when skin-sparing mastectomy was performed. Large skin island marks the remaining skin of the flap formed during reconstruction following simple or modified radical mastectomy, or secondary reconstruction. Small skin island was evaluated as an individual site. Large skin island area was divided into five regions as illustrated in Figure 1. Unoperated contralateral breasts were examined in a similar manner. Besides the regions previously labeled 1–4, for the central control region, only the immediate periareolar skin was tested due to the different composition of mechanoreceptors in the nipple and areola skin [26].

Static tactile-pressure perception thresholds were measured using the set of five Semmes-Weinstein type monofilaments: 6.65, 4.56, 4.31, 3.61, and 2.83 (Baseline^®^Tactile monofilament evaluator, Fabrication Enterprises Inc., USA). The corresponding monofilament marking signifies a logarithm of 10 times the force in milligrams necessary to bow it. Monofilaments were applied perpendicularly to the skin and pressed to bend. When a subject perceived the stimulus, a positive response was recorded [2]. In the first testing sequence, monofilaments were introduced in descending order to screen for the level of perceivable sensation. Subsequently, an adaptive procedure depending on the previously detected thresholds was used. The monofilaments exerting forces adjacent to the thinnest perceived were presented in a random series to avoid anticipation bias. Every region was tested until at least three positive responses were obtained or up to a maximum of five times if the stimulus could not be detected. The thinnest monofilament detectable three times in an individual area was considered the representative. Testing order of the different regions was also random. Throughout the sensory testing, subjects were lying on the examination table and had their eyes closed. The examination was conducted in a quiet room by the first author.

### Data interpretation and analysis

Considering that every monofilament in the set belongs to a certain category of sensation [25], the representative monofilament for each tested area was scored on a 1–6 scale as follows: 1 – without measurable sensation, 2 – 6.65, 3 – 4.56, 4 – 4.31, 5 – 3.61, and 6 – 2.83 [3], 4]. Furthermore, to assess flap skin island and healthy breast sensation more accurately, three sensory scores were calculated: mean regional, peripheral, and total sensory score. Mean regional sensory score (MRSS) was defined as an arithmetic mean of the tactile perception values (rated 1–6) for a single region of the flap skin or healthy breast. Peripheral sensory score was calculated as a sum of MRSSs of the four peripheral skin regions in large skin islands, whereas a sum of all five MRSSs was named total sensory score. For small skin islands, mean regional equaled total sensory score. The advantage of the created sensory scores is that they simultaneously indicate level, frequency, and for large skin islands also extent of tactile sensation recovery.

Due to the small sample size, limited statistical analysis was performed. Descriptive statistics including frequencies, medians with ranges, and means with standard deviations was used to describe subjects’ characteristics and sensory recovery parameters. Using MedCalc^®^ statistical software [27], Fisher’s exact test was performed to compare the occurrence of sensory recovery between the IMMUNO+ and IMMUNO− subgroups for every large skin island region. p-value <0.05 was considered statistically significant.

## Results

### Subjects’ characteristics

Seventeen out of 25 patients altogether were available for the study. Three patients were excluded from the analysis, two not having the exposed flap skin, and one operated due to polymastia. Therefore, 14 patients operated due to malignant breast tumor with 14 MS TRAM flap breast reconstructions were included. Nine of them presented with large skin island, while the rest five had small skin island. Vascular supply of the flap was established via anastomoses with internal mammary artery and vein in all subjects. Not a single subject had a nipple-areola complex reconstruction, a history of diabetes mellitus, peripheral polyneuropathy, or neurological disease affecting sensory perception. Other relevant subjects’ data are listed in Table 1.

**Table 1:** Subjects’ characteristics concerning age, body mass index, breast cancer treatment, and reconstruction.

MS TRAM free flap skin island size groups	Large skin island (n=9)		Small skin island (n=5)	
Immunotherapy status subgroups	**IMMUNO−**	**IMMUNO+**	**IMMUNO−**	**IMMUNO+**
Number of subjects, %	6 (67 %)	3 (33 %)	4 (80 %)	1 (20 %)
Median age (range)	59.5 (30–71)	56.0 (42–66)	55.0 (53–58)	57.0
Mean body mass index, SD	28.1 (2.4)	29.3 (4.5)	27.1 (3.6)	31.1
Median time interval from reconstruction to examination in months (range)	39.5 (5–61)	28.0 (16–45)	34.0 (26–49)	6.0
Primary reconstruction	2	1	4	1
Secondary reconstruction^a^	4	2	0	0
Radiotherapy	1	0	0	1
Chemotherapy^b^	2	3^c^	1	1
Endocrine therapy	3	3	3	1
Axillary lymph node dissection	5	1	1	0
Diabetes mellitus	0	0	0	0

^a^Two subjects from IMMUNO− and two subjects from IMMUNO+ large skin island subgroups previously had implant-based breast reconstruction. ^b^Five subjects received 4AC-12P chemotherapy regimen (doxorubicin, cyclophosphamide, paclitaxel). ^c^For one patient, data on chemotherapeutic agents were not available, one patient received multiple chemotherapeutic regimens due to recurrent disease.

Four patients in total were administered immunotherapy with trastuzumab (HERCEPTIN, Genentech, South San Francisco, CA). One of them was receiving a combination with pertuzumab (PERJETA, Genentech, South San Francisco, CA). Another of them was treated with ado-trastuzumab emtansine (KADCYLA, Genentech, South San Francisco, CA) following the completion of trastuzumab regimen.

### Large skin island sensory recovery

**Figure 2:**
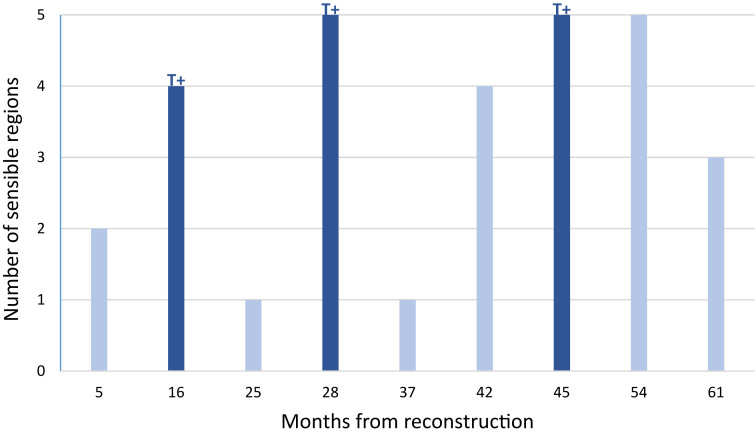
The number of large skin island regions with detectable tactile sensation in each subject. Subjects treated with immunotherapy are marked with T+.

All subjects with large skin island (n=9) regained measurable tactile sensation in at least one tested region. The recovery of all five regions was seen only in three participants, two of which had received immunotherapy. Number of sensible regions in each subject with large skin island is shown in Figure 2. It is of note that more extensive recovery (four to five sensible regions) was seen already at 16 months in the IMMUNO+ subjects, whereas in the IMMUNO− subgroup, the same extent was recorded after 42 months.

**Figure 3:**
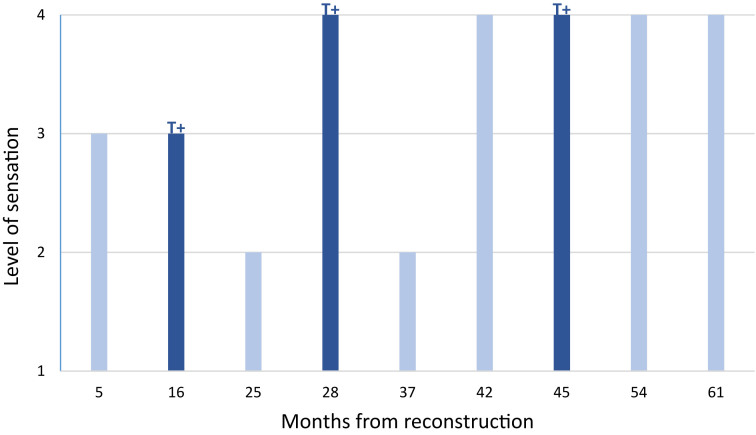
The maximum level of sensation detectable in at least one large skin island region in each subject. Levels of sensation: 1 – no measurable sensation, 2 – monofilament 6.65, 3 – monofilament 4.56, and 4 – monofilament 4.31. Subjects treated with immunotherapy are marked with T+.

All subjects who were treated with immunotherapy recovered tactile sensation to the central region of large skin island, opposed to only one subject in the IMMUNO− subgroup with the sensible central area 54 months following reconstruction (p=0.0476). The occurrence of detectable tactile sensation in the peripheral regions was not shown to be statistically significant (superior medial, p=0.1667; inferior medial, p=1; inferior lateral, p=0.5; superior lateral, p=1). The thinnest monofilament perceivable in subjects with large skin island was monofilament 4.31. It was earliest detected at 28 months in the IMMUNO+ and 42 months in the IMMUNO− subgroup. In both of these subjects, it extended to three of five tested regions. Nevertheless, the respective level of sensation was equal to the highest tactile threshold in the contralateral healthy breasts. Figure 3 illustrates the maximum level of sensation detectable in at least one tested region in each subject.

Finally, the IMMUNO+ subjects appeared to recover sensation more consistently than the IMMUNO− ones. Total sensory score for the IMMUNO+ reconstructed breasts was 54 % of the control reference breast in comparison to 39 % in the IMMUNO− breasts. Table 2 demonstrates the results of monofilament examination for a particular region as well as the previously introduced sensory scores.

**Table 2:** Results of monofilament examination and calculated sensory scores (mean regional, peripheral, total) in the regions of large skin islands and healthy contralateral breasts. There is a sustainable trend of superior mean regional sensory scores for every large skin island region in subjects treated with immunotherapy. In both experimental subgroups, three monofilaments were perceivable (6.65, 4.56, 4.31).

Tested region of the large skin island	IMMUNO− (n=6)	IMMUNO+ (n=3)	Healthy contralateral breasts (n=5)
**Superior medial**			

MMV^a^, SD	4.31 (0)	5.17 (1.28)	3.44 (0.62)
% of sensible regions	33.33 %	100 %	100 %
MRSS^b^	2.00	3.00	5.20

**Inferior medial**			

MMV^a^, SD	4.93 (0.97)	4.39 (0.14)	3.44 (0.62)
% of sensible regions	83.33 %	100 %	100 %
MRSS^b^	2.67	3.67	5.20

**Inferior lateral**			

MMV^a^, SD	5.02 (1.09)	5.17 (1.28)	3.30 (0.43)
% of sensible regions	66.67 %	100 %	100 %
MRSS^b^	2.33	3.00	5.40

**Superior lateral**			

MMV^a^, SD	6.13 (1.05)	5.61 (1.48)	3.30 (0.43)
% of sensible regions	66.67 %	66.67 %	100 %
MRSS^b^	1.83	2.00	5.40

**All peripheral regions**			

MMV^a^, SD	5.19 (1.07)	5.04 (1.04)	3.37 (0.50)
% of sensible regions	61.50 %	91.67 %	100 %
Peripheral sensory score	8.83	11.67	21.20

**Central**			

MMV^a^, SD	4.31 (0)	5.87 (1.35)	3.44 (0.43)
% of sensible regions	16.67 %	100 %	100 %
MRSS^b^	1.50	2.67	5.20

**Entire flap/breast skin**			

MMV^a^, SD	5.14 (1.06)	5.22 (1.11)	3.38 (0.48)
% of sensible regions	53.33 %	93.33 %	100 %
Total sensory score	10.33	14.34	26.40

^a^MMV, mean monofilament value (arithmetic mean of the perceivable representative monofilaments). ^b^MRSS, mean regional sensory score.

### Small skin island sensory recovery

In the group of subjects with small skin island (n=5), all but one recovered measurable tactile sensation in the single tested region. The subject without perceivable sensation had her breast reconstructed 34 months prior to the examination and was not administered immunotherapy. The only IMMUNO+ subject restored tactile sensibility to the values of her contralateral periareolar breast skin as early as 6 months postoperatively. The IMMUNO− subject tested 26 months after reconstruction also retrieved sensibility equal to the contralateral control area. The thinnest monofilament detectable in small skin islands was 4.31 same as it was in large skin islands. Table 3 displays the results of monofilament testing and calculated total sensory scores. Relative to the reference healthy breast score, the IMMUNO− subjects recovered 71 %, while the IMMUNO+ subject reached 95 % of the control sensory score.

**Table 3:** Results of monofilament examination and calculated total sensory scores in small skin islands and healthy contralateral breasts.

Single tested region of the small skin island	IMMUNO− (n=4)	IMMUNO+ (n=1)	Healthy contralateral periareolar skin (n=5)
MMV^a^, SD	4.39 (0.14)	4.31 (0)	4.08 (0.44)
% of sensible regions	75 %	100 %	100 %
Total sensory score	3.00	4.00	4.20

^a^MMV, mean monofilament value (arithmetic mean of the perceivable representative monofilaments).

### Immunotherapy treatment timing relative to mastectomy and reconstruction

To comprehend the potential mechanisms of trastuzumab’s actions on nerve regeneration, the treatment course will be described meticulously in the four respective subjects. Three subjects were administered a regimen of 17 applications of trastuzumab given every 3 weeks. First of them had started immunotherapy a month after primary autologous breast reconstruction, and 45 months later she regenerated tactile sensation to all large skin island regions. Second of them had received seven applications of trastuzumab preoperatively and 10 applications following primary autologous breast reconstruction. During four initial immunotherapy applications, both trastuzumab and pertuzumab were given. She recovered sensation in small skin island to the level of her contralateral breast. Third of the subjects had started trastuzumab treatment immediately after primary implant-based breast reconstruction. Due to capsular contracture, implant was extracted and the breast reconstructed with free flap. Trastuzumab immunotherapy was finalized 1 month after autologous reconstruction. She regenerated sensation to four large skin island regions including the central one 16 months postoperatively. The fourth subject underwent autologous breast reconstruction secondary to development of cutaneous metastasis on top of implant-based reconstruction done 6 years before. The subject was treated with trastuzumab immunotherapy multiple times. Initially, she was receiving trastuzumab for a year starting 5 months after subcutaneous mastectomy and primary implant-based breast reconstruction. Secondly, she had received 18 applications of trastuzumab during a year and half prior to free flap reconstruction. Lastly, 4 months after autologous reconstruction, she had started a course of six applications of trastuzumab followed by four applications of ado-trastuzumab emtansine. She recovered sensation to all large skin island regions 28 months postoperatively.

## Discussion

The present study for the first time indicated that trastuzumab may be able to improve sensory reinnervation in free flap breast reconstructions. Subjects who were receiving trastuzumab recovered touch sensation earlier and more consistently. The probable underlying mechanisms of trastuzumab’s effects on peripheral nervous system regeneration and comparison with the existing data on sensory recovery in noninnervated and innervated TRAM and DIEP flaps are discussed below.

### Probable mechanisms of trastuzumab’s effects on peripheral nerve regeneration

Trastuzumab is a monoclonal antibody that specifically binds to the subdomain IV of the human epidermal growth factor receptor 2 (HER2) [20]. HER2 (ErbB2) belongs to the ErbB family of transmembrane tyrosine kinases receptors, along with the epidermal growth factor receptor (EGFR, ErbB1), ErbB3, and ErbB4 [28]. Whereas numerous peptide ligands act as agonists for the other three receptors [29], ErbB2 itself has no known ligand but is the preferential heterodimerization partner of the other members [30]. The aforementioned animal studies, which evidenced positive effects of trastuzumab on peripheral nerve regeneration, evaluated a few potentially involved signaling pathways [23], 24]. Hendry et al. hypothesized that the blockade of EGFR/ErbB2 dimer formation by trastuzumab might be the underlying mechanism, since EGFR was recognized as an inhibitor of axonal regeneration. Indeed, trastuzumab was shown to significantly reduce phosphorylated EGFR activity on neurons proximal to the repair site [24]. Histomorphologically, trastuzumab-treated rats regenerated significantly higher number of myelinated axons in distal nerve stumps 4 weeks after immediate repair compared to saline-treated animals while myelin thickness remained preserved [23], 24]. Preceding, there were significantly more motor and sensory neurons regenerating their axons a week after injury. Beyond the effects on neurons, trastuzumab appeared to increase distal proliferation of Schwann cells and macrophages [24]. In addition, a recent study by Topley et al. indicated that EGFR inhibitor gefitinib may raise the rate of sensory regeneration early after median nerve transection in mice [31].

In accordance with the reported findings of animal studies, there are at least two explanations on how trastuzumab could improve sensory reinnervation in our subjects. First is the enhancement of axonal sprouting [23] that we find especially beneficial in the circumstances of spontaneous reinnervation in free flaps. More abundant sprouting could help axons from recipient bed and surrounding skin to more easily find distal pathway in a foreign neurilemmal network of transferred tissue. Indeed, skin biopsies from myocutaneous noninnervated flaps indicated that regenerating axons are most likely guided by the preexisting empty neurilemmal sheaths [32]. Secondly, more rapid axonal elongation supported by trastuzumab was suggested to promote distal cellular proliferation due to sooner axonal contact with denervated Schwann cells [24]. Since chronically denervated distal nerve stumps gradually weaken their growth-supportive capacity due to the progressive fading of Schwann cell repair phenotype and loss of Schwann cells [33], trastuzumab-accelerated axonal regeneration could stimulate growth-encouraging environment in distal nerve stumps and thereby improve target reinnervation.

Another explanation is that trastuzumab could play a protective role against the paclitaxel neurotoxicity. In their population-based cohort study on taxane-induced peripheral neuropathy, Patel et al. observed lower incidence of neuropathic pain medication first prescription in patients treated with paclitaxel plus trastuzumab compared to paclitaxel only regimen [34]. We assume this possibility could only have a minor influence since far less subjects in the IMMUNO− subgroups (3 out of 10) were treated with chemotherapy.

The two other hypothetical possibilities of trastuzumab’s actions encompass a protective activity against neuronal cell loss [35] in dorsal root ganglia (DRG) after nerve transection and eventual functional reconstruction in DRG mediated by sprouting. First of the possibilities could serve a particularly interesting role in maintaining a larger pool of DRG neurons surviving long-term after mastectomy, which may positively affect the reinnervation capacity in secondary free flap breast reconstructions. Further experimental studies are necessary to elucidate effects of trastuzumab at potentially multiple sites of action, including DRG neurons and satellite glial cells, different populations of Schwann cells, sensory corpuscles, and non-neuronal cells.

### Comparison with the previous studies on sensory recovery after free flap breast reconstruction

In the current study, all except one subject with MS TRAM flap small skin island regenerated tactile sensation to the levels closely comparable to ones of the contralateral periareolar skin. The maximum level of sensation (monofilament 4.31) was earliest detected in subjects with the shortest intervals from reconstruction in both small skin island subgroups, at 6 months in the IMMUNO+ and 28 months in the IMMUNO− one. On the contrary, in the large skin island group, monofilament 4.31 was perceivable after 28 months in the IMMUNO+ and 42 months in the IMMUNO− subjects. These observations are related to the previous report on earlier spontaneous reinnervation in smaller DIEP flaps [9]. In addition, we noticed that more extensive sensory recovery, involving at least four of five large skin island regions, occurred in all subjects treated with trastuzumab (at the earliest after 16 months), whereas in subjects who did not receive immunotherapy, it was evident during later post-reconstruction period (at the earliest after 42 months). Similarly, Stromps et al. found that sensory recovery in three or more segments of DIEP flaps started from the 30th postoperative month while testing tactile and other modalities [9].

The three subjects with MS TRAM flap large skin island who were receiving trastuzumab recovered 54 % of the reference breast total sensory score after averagely 2 and half years. Puonti et al. reported the restoration of 45 % of the contralateral total sensory score (including tactile, thermal, vibratory, and noxious modalities) in MS TRAM flaps with nerve repair at an average of 2 years after delayed reconstruction [14]. Although these ratios are not directly comparable due to the different contributions of various tested modalities in Puonti’s study [14], they imply that trastuzumab administration in noninnervated MS TRAM flaps could lead to at least similar overall recovery of tactile sensation as in innervated MS TRAM flaps. In our study, mean regional sensory scores were calculated the same way as mean Semmes-Weinstein scores in the study by Blondeel et al. [4]. Scores for the five regions of noninnervated TRAM flaps (1.39–2.32) in their study [4] are in similar range as obtained in our subjects who were not treated with immunotherapy (1.50–2.67). Furthermore, mean regional sensory scores for the large skin island subgroup receiving trastuzumab in the current study (2.00–3.67) are slightly lower than scores in their group of DIEP flaps with nerve repair (2.46–3.58), which was comprised of both primary and secondary breast reconstructions where not only flap skin was tested [4]. Regarding the level of sensation, mean monofilament value for the entire MS TRAM flap large skin island in trastuzumab-treated subjects was 5.22±1.11, which is similar as in noninnervated DIEP flaps (5.52±0.55) but poorer than in innervated DIEP flaps (4.17±0.70) as noted by Beugels et al. [17].

Contrary to the frequently documented poor or absent sensation in the central area of noninnervated TRAM [5], 11], 13] and DIEP [4], 6] flaps, all subjects in our study who were treated with trastuzumab recovered tactile sensation to the detectable levels in the central flap region. Considering the previously described pattern of reinnervation in noninnervated flaps starting peripherally and progressing inwards [4], 6], it can be concluded that the restoration of sensation centrally is an indicator of advanced sensory reinnervation in flaps without nerve repair. Finally, subjects treated with trastuzumab appeared to overcome the unpredictability and inconsistency of sensory recovery in TRAM flaps that was seen in our subjects who were not administered immunotherapy as well as reported earlier [1], 4]. It remains to be explored if patients with noninnervated and innervated DIEP flaps receiving trastuzumab can regain sensation closer resembling one of the healthy breasts. Remarkably, effects of HER2- and EFGR-targeted drugs on peripheral sensory nerve regeneration could be examined in autologous breast reconstructions, potentially paving the way to their local application in injured peripheral nerves.

### Limitations of the study

We understand this is a small study with limited number of subjects that disabled proper statistical analysis. This is because initially we did not expect to subdivide subjects with respect to immunotherapy administration. Another limitation is the diversity of breast cancer treatment received by participants that can hardly be avoided in observational clinical study setting. Additional studies involving more participants and evaluating sensory modalities other than tactile in a prospective manner are needed to draw definite conclusions.

## Conclusions

Immunotherapy treatment stands out as a novel factor affecting sensory outcome after autologous breast reconstruction. To the authors’ knowledge, these are the first observations on accelerated and more consistent spontaneous sensory recovery after MS TRAM free flap breast reconstruction in patients treated with trastuzumab. Further studies are necessary to elucidate the observed effects and potentially broaden application of available HER2 and EGFR inhibitors to regenerate neural tissues. Henceforth, immunotherapy should be given greater importance while evaluating new strategies for enhancement of sensory recovery in reconstructed breasts.
